# Residents’ Perspectives on Graduate Medical Education during the COVID-19 Pandemic and Beyond

**DOI:** 10.15694/mep.2020.000077.1

**Published:** 2020-04-27

**Authors:** William Rainey Johnson, David Blitzer

**Affiliations:** 1 Walter Reed National Military Medical Center; 2 Columbia University

**Keywords:** Graduate medical education, COVD-19, Simulation, Virtual Reality, Telehealth, Scarcity

## Abstract

This article was migrated. The article was marked as recommended.

Graduate medical education (GME) programs are saddled with the dual responsibilities of exceptional healthcare delivery, while ensuring their trainees’ specialty-specific competency. The COVID-19 pandemic threatens this dual mission. The scarcity of resources has required redistribution of personnel, including trainees, and limitations on the number of personnel interacting with patients. These changes have lowered specialty specific clinical volume for trainees. GME programs must look for new ways to educate trainees. Failure to do so may lead to a bottleneck within the medical education training pipeline or graduation of less than fully competent physicians. As two GME trainees on the frontlines, we describe the negative impacts of the COVID-19 pandemic on current GME training in the United States. We then propose possible remedies to the problem. To account for lost training, we discuss potential solutions for filling gaps in training and, simultaneously, urge a coordinated effort among leaders in GME to use the pandemic to catalyze a revolution that will improving training now and in the future.

## Background and Purpose

The COVID-19 pandemic impacts every part of society. While the healthcare system serves on the frontlines of the COVID-19 pandemic, graduate medical education (GME) in the United States has suffered. While we focus our discussion on GME in the United States, we suspect that the omnipresence of the COVID-19 pandemic globally has or will have similar impacts everywhere. GME programs are saddled with the dual duties of exceptional healthcare delivery and providing trainees’ specialty specific competency growth. As GME trainees, we are acutely aware of the evolution toward competency-based GME and recognize that amid the challenges of the current pandemic, is also the opportunity to redouble our collective efforts to train justifiably competent physicians.

The medical profession has an ethical obligation to treat, prioritizing public health and patient safety during the pandemic. Similarly, GME has an ethical obligation to prepare physicians for competent independent practice. In the face of the pandemic, therefore, we must acknowledge the potentially adverse consequences for medical trainees. While this trend has been addressed in medical student education, the effects of the pandemic on GME have not been described ( Goh and Sandars, 2020; Rose, 2020). Below, we delineate the negative impacts of the COVID-19 pandemic on current GME training in the United States, based on our experiences as GME trainees in different programs and specialties (cardiothoracic surgery and internal medicine), and offer potential recommendations to accommodate resident education during these unprecedented times. In doing so, we hope to ignite a change in GME that will have a positive impact for trainees within and beyond the pandemic, while also encouraging all trainees to uphold their societal obligations to serve and treat patients in this time of need.

## Negative Impact of COVID-19 Pandemic on GME Training

The COVID-19 pandemic has affected clinical and non-clinical GME. To reduce the risk of unnecessary exposure and to concentrate limited (including human) resources, healthcare systems have stopped elective surgeries and outpatient appointments. As trainees, our rotations rely on these services and so have also stopped. The critical shortage of personal protective equipment has also decreased GME operating room time and face-to-face clinical learning. Future attending physician shortages may further limit the development of junior trainees, who generally require more supervision and so are more likely to be exclude in a minimal-contact environment. Diminished clinical volume in operating rooms wards reduces opportunities for surgical and non-surgical trainees to develop clinical practice. These adaptive measures, while important for patient care, may stunt professional development, threatening trainees’ preparedness, and weaken innovative leadership during future pandemics when today’s trainees will be the decision makers.

The impact of the COVID-19 pandemic on GME will continue and expand. Surges in patient volumes requiring treatment for COVID-19, coupled with essential infection control precautions, will continue to have severe consequences on GME training. Many of our peers have been, and will continue to be, quarantined, requiring weeks away from direct patient care. Other peers have and will continue to be redeployed to duties outside their specialty care, temporarily eliminating educational experience within their selected specialty. Still other peers are stationed in reserve at home, waiting to fill gaps in essential coverage. All of these factors, while necessary during a national emergency, reduce trainees’ competency development by limiting the cornerstone of GME training: work-place based learning and apprenticeship.

The need to limit human-to-human exposure. required changes in trainee non-clinical education time. In-person conferences have dissolved, but this provides an opportunity for more accessible offerings. Some programs have leveraged established video lecture series or board-preparation curricula as a replacement. Our programs have implemented e-learning technology to have synchronous and asynchronous virtual conferences, an approach gaining prevalence even before the pandemic ( Wittich *et al.*, 2017). These alone, however, cannot replace the volume of clinical encounters lost because of the changes required by COVID-19.

## GME Training Must Adapt

Training programs must consider how they can replace in-person clinical experiences to prevent delays in graduation and/or competency gaps. Failure of GME to rapidly adapt will contribute to the growing physician shortage due to a backup in the training pipeline or influx of less than fully competency physicians, jeopardizing future patient safety ( Dall *et al.*, 2019). As a result, GME cannot just wait out the pandemic. GME programs have successfully responded to disasters in the past, delivering exceptional patient care while continuing to educate effectively their trainees ( Davis, 2006; Espana-Schmidt *et al.*, 2013). Then, regional solutions were options. After Hurricanes Katrina and Sandy, staff and trainees volunteered to meet the brief increased patient demand, while regional healthcare systems absorbed longer term patient and trainee surpluses ( Davis, 2006; Espana-Schmidt *et al.*, 2013). In resource-poor countries, GME programs have had less success. During the West African Ebola epidemic, local GME training programs closed, despite efforts from geographically separated healthcare systems to provide distance learning, personal protective equipment, and healthcare workers ( Mcquilkin *et al.*, 2017). The geographic ubiquity, prevalence of disease, and expected duration of the current pandemic is unprecedented. Yet these historical events present some critical elements for a successful GME response. A pandemic of this magnitude requires a comprehensive and coordinated effort to accomplish the GME mission, including delivery of excellent patient care, at a local, regional, and national scale.

## Potential Solutions to Minimize Negative Training Impact During the Pandemic and Beyond

Interactions with real and virtual patients could help fill the anticipated training gaps. Rapid implementation of telehealth would allow for many meaningful clinical interactions without potential viral exposure and would better prepare trainees for the future integration of tele-communications in healthcare ( Der-Martirosian *et al.*, 2019; Zimlichman *et al.*, 2019). In response to this pandemic, many healthcare systems have and continue to increase implementation of telehealth services, which will likely further augment future telehealth use. Virtual patients may also have potential. For procedural specialties, this may include simulation experiences or virtual reality which have already been implemented across several specialties ( Borgersen *et al.*, 2018; Gideon, 2019). One could imagine similar technology in non-procedural specialties through partnerships with video gaming companies to create virtual clinical experiences that would require knowledge, skills, and attitudes aligned with accredited competency domains. Regardless of the GME evolution during the pandemic, training programs nationally, and even internationally, must openly communicate; sharing approaches and resources will help ensure that all trainees have equal opportunity to succeed in a resource constrained time.

Even after the pandemic, such educational advances could supplement and complement clinical practice and exposure. It is not hard to imagine that, by increasing flexibility and autonomy, these same efforts could be parlayed into tools to address other limitations of the GME status quo: for example, increasing the duration of parental leave, catching-up from an unexpected leave of absence, or improving standardized competency assessments. Many licensing and board certifying standardized examinations have strong validity evidence for medical knowledge domains, while lacking significant evidence in other, equally important, competency domains ( Babbott *et al.*, 2007; Kay, Jackson and Frank, 2015). As a result, despite the move toward competency-based training, GME remains a time-dependent training pipeline. Development of higher fidelity, virtual platforms, may revolutionize how we assess clinical competence, allowing GME to more fully embrace a time-independent, standardized competency-based educational model. In such a model, GME could justifiably graduate competent physicians, while assigning additional training to less than fully competent physicians. GME would be better prepared for future interruptions and drastic changes, like the COVID-19 pandemic, and more flexible and inclusive to the needs of individual trainees.

## Conclusion

The healthcare system faces unprecedented challenges in the COVID-19 pandemic. We understand that the changes and limitations imposed on GME are necessary and just and we urge every individual medical trainee to do his or her part. As trainees, we will experience the realities of contagion control, rationing, and crisis response and gain real world experience in epidemiology, public health, public policy, ethics, and even palliative care. These experiences will no doubt be formative and transformative, inspiring us and our peers to become the leaders of today and tomorrow. But, as trainees, our clinical progression and competence need not suffer. We remain optimistic that the pandemic will catalyze a healthcare revolution to deliver more patient-centered care. Simultaneously, as trainees, we urge GME leaders to seize upon this moment with a coordinated effort to create a parallel revolution in GME, which will ultimately benefit the trainees of today and patients of tomorrow.

## Take Home Messages

• Physicians, including graduate medical education (GME) trainees, have an ethical obligation to prioritize public health and safety during the pandemic
• GME must also uphold its obligation to train competent physicians
• Changes to GME training during the unprecedented COVID-19 pandemic threaten trainee preparedness for competent independent practice
• Trainee preparedness could be maintained during the COVID-19 pandemic by creating more opportunities for real and virtual patient interactions using telehealth platforms and simulation/virtual reality, and creating educational best practices for each of these
• More than just a temporary obstacle, the COVID-19 pandemic could catalyze a revolution in GME training: we urge leaders to seize the moment in a coordinated effort

## Notes On Contributors

W. Rainey Johnson is a second-year resident in internal medicine at Walter Reed National Military Medical Center. He earned his Doctor of Medicine at the University of Pennsylvania and his Master of Education (curriculum and instruction) at the University of Nevada - Las Vegas. He is currently pursuing a Doctor of Philosophy in Health Professions Education. His ORCID is https://orcid.org/0000-0001-8982-4126.

David Blitzer is a third-year resident in cardiothoracic surgery at Columbia University. He earned his Doctor of Medicine and Master of Bioethics at the University of Pennsylvania.
