# Supplementary material for: Residents’ Perspectives on Graduate Medical Education during the COVID-19 Pandemic and Beyond
Source: MedEdPublish (2016). 2020 Apr 27;9:77. [Version 1] doi: 10.15694/mep.2020.000077.1 (PMC10697462; doi:10.15694/mep.2020.000077.1)
Supplement: Peer review history

# Reviewer report: Reviewer response for version 1

**DOI:** 10.21956/mep.20041.r30688

**Published:** 2020-11-28

**Authors:** Ken Masters

**Affiliations:** 1 Sultan Qaboos University

This review has been migrated. The reviewer awarded 4 stars out of 5 An interesting paper on residents’ perspectives on Graduate Medical Education during the COVID-19 pandemic and beyond. The authors draw on experience and lessons learned from several other emergency situations, and propose strategies for coping with COVID-19It is interesting to read this paper with the hindsight of what has passed in the time since the paper’s first publication. Among the interesting issues are the predictions of the impact of the pandemic on GME, and these appear to have been accurate.It remains to be seen whether or not the warnings for the future will be heeded. One hopes so, but, unfortunately, complacency is a great enemy.An interesting read, indeed.

Reviewer Expertise:

NA

No decision status is available

# Reviewer report: Reviewer response for version 1

**DOI:** 10.21956/mep.20041.r30689

**Published:** 2020-11-15

**Authors:** Barbara Jennings

**Affiliations:** 1 Norwich Medical School

This review has been migrated. The reviewer awarded 4 stars out of 5 In this clear and interesting article, the authors explore the challenges created by the current need to limit human exposure because of covid-19, and the implications for both the graduate trainee and healthcare systems during and beyond the pandemic. The authors are residents in surgery and medicine and I think their perspective will make an interesting read for trainees and graduate programme leads in many settings globally.The authors comment on events that can be learned from in 2020 with lessons from earlier epidemics crises; and they cite some valuable literature. Furthermore, ideas for adapting and developing graduate medical education are outlined (e.g. with expansion of telehealth and the importance of the virtual patient) which resonate with suggestions discussed in a recent journal commentary (Hays, R. et al. MedEdPublish, 9, [1], 142, https://doi.org/10.15694/mep.2020.000142.2) that cited this article by Johnson and Blitzer.My expectation is that many of the useful ideas proposed here and by others (about technology enhanced practice) will trigger research and innovative pilot programmes in institutions across the world.

Reviewer Expertise:

NA

No decision status is available

# Reviewer report: Reviewer response for version 1

**DOI:** 10.21956/mep.20041.r30686

**Published:** 2020-04-28

**Authors:** Samar Ahmed

**Affiliations:** 1 Ain Shams University Faculty of Medicine

This review has been migrated. The reviewer awarded 3 stars out of 5 I want to thank the author for taking time to use a positive lens to dissect the current challenges. The use if telemedicine to overcome the training problems with distancing is a very important approach. I would have loved to see a reflection of the author himself on his own experience and maybe a study demonstrating how the experience worked for him

Reviewer Expertise:

NA

No decision status is available

# Reviewer report: Reviewer response for version 1

**DOI:** 10.21956/mep.20041.r30687

**Published:** 2020-04-27

**Authors:** Sarah Fairbrook

**Affiliations:** 1 MedStar Washington Hospital Center

This review has been migrated. The reviewer awarded 5 stars out of 5 Reframing the crisis as an opportunity in education is an important challenge that should be embraced by all health professions educators. The authors offer substantive ideas for moving forward balancing the need for competent clinicians and the ethical demands of a global pandemic.

Reviewer Expertise:

NA

No decision status is available
